# Neurological Soft Signs in Schizophrenia, a Picture of the Knowledge in the Last Decade: A Scoping Review

**DOI:** 10.3390/healthcare11101471

**Published:** 2023-05-18

**Authors:** Cristian Petrescu, Diana M. Petrescu, Gabriela Marian, Brindusa E. Focseneanu, Floris Petru Iliuta, Constantin Alexandru Ciobanu, Serban Papacocea, Adela M. Ciobanu

**Affiliations:** 1 Department of Psychiatry, Faculty of Medicine, Carol Davila University of Medicine and Pharmacy, 020021 Bucharest, Romania; cristian.petrescu@drd.umfcd.ro; 2 Department of Psychiatry, Prof. Dr. Alexandru Obregia Clinical Hospital of Psychiatry, 041914 Bucharest, Romania; brindusafocseneanu@yahoo.com; 3 Neurology Clinic Fundeni Clinical Institute, 022328 Bucharest, Romania; diana-mihaela.vlad@rez.umfcd.ro; 4 Academy of Romanian Scientists, 050045 Bucharest, Romania; gabi.marian@yahoo.com; 5 Department of Psychiatry and Psychology, Titu Maiorescu University of Medicine, 040441 Bucharest, Romania; 6 Department of Psychiatry and Psychology, Carol Davila University of Medicine and Pharmacy, 020021 Bucharest, Romania; florisiliuta@gmail.com; 7 Faculty of Medicine, Titu Maiorescu University of Medicine and Pharmacy, 040441 Bucharest, Romania; 8 Department of Neurosurgery, Faculty of Medicine, Carol Davila University of Medicine and Pharmacy, 020021 Bucharest, Romania; serban.papacocea@drd.umfcd.ro

**Keywords:** neurological soft signs, schizophrenia, rating scales, review

## Abstract

(1) Background: Neurological Soft Signs (NSS) are subtle neurological abnormalities that are more common in schizophrenia patients than in healthy individuals and have been regularly observed in neuroleptic-naive first-episode patients, supporting the hypothesis that they are an intrinsic component of schizophrenia. (2) Methods: a review of articles published in the last ten years (from January 2013 to January 2023) was carried out on articles published in ScienceDirect and PubMed, by following the PRISMA Statement extension for scoping reviews (PRISMA-ScR), which evaluated the impact of NSS in correlation with the symptomatology, neuroleptic treatment, and the cerebral structural changes of patients with schizophrenia. (3) Results: thirty articles were included, among them twelve included MRI structural evaluation and four studies with a longitudinal design. (4) Conclusions: interest in researching NSS has increased in recent years, but questions remain about their origin and relationship to schizophrenia symptoms, thus this study aims to fill in information gaps in the hope that future research will help provide individualized treatment. It is suggested that NSS in schizophrenia might have an inherited genetic relationship pattern, thus being in line with a trait viewpoint. Most of the research revealed that schizophrenia patients had higher NSS scores than healthy controls, however, they were rather similar to their first-degree relatives, thus, also arguing in favor of a trait perspective. The greatest improvement in scores is seen in those with a remitting course, as shown by declining NSS ratings concurrent with symptomatology.

## 1. Introduction

Schizophrenia is a chronic and severe neuropsychiatric disorder that markedly affects cognition, perception, emotions, and conduct, leading to reduced quality of life and disability [1]. Negative symptoms, which are a prominent characteristic of schizophrenia, are a major contributor to long-term morbidity and suboptimal functional outcomes [2,3]. The treatment of schizophrenia typically involves antipsychotic medication, which can help alleviate positive symptoms such as hallucinations and delusions [4]. The choice of medication and dosage will depend on the individual’s symptoms and response to treatment. Antipsychotic medications can have side effects, such as weight gain, drowsiness, and neurological impairment [5]. Psychotherapy, social support, and lifestyle changes such as regular exercise, a healthy diet, and adequate sleep can also be beneficial in managing symptoms and improving overall well-being [6].

It has long been recognized that motor dysfunction is a core characteristic of schizophrenia and other spectrum illnesses [7]. Neurological soft signs, commonly known as NSS, are subtle neurological abnormalities that include difficulties with sensory integration, motor coordination, and sequencing of complex motor actions, as well as abnormalities with eye movements and neurodevelopmental reflexes [8,9,10].

These neurological abnormalities are not caused by hard pathologies, such as regional, nuclear, or primary tract lesions, and the irregular movements, which resemble tardive dyskinesia, are not side effects of using neuroleptic drugs but rather a feature of the disease itself [11,12,13,14]. One of the earliest pieces of evidence comes from early clinical observers who, before the development of antipsychotic drugs, noted motor impairment in schizophrenia patients [12,15]. It is well acknowledged that NSS are more common in schizophrenia patients than in healthy individuals [16,17]. They have been regularly observed in neuroleptic-naive first-episode patients, supporting the hypothesis that NSS are an intrinsic component of schizophrenia [7,18]. Increased NSS scores in high-risk subjects, such as relatives of schizophrenia patients or unaffected twins, support this hypothesis [19,20,21,22,23]. NSSs are considerably more prevalent in patients with schizophrenia than in their first-degree relatives, and they are more prevalent in first-degree relatives than in healthy controls [18,24]. NSS scores in patients and their relatives are comparable and they influence motor coordination, motor sequencing, and sensory integration [14]. NSS usually decreases in patients who respond well to medication, while it persists or increases in individuals who do not respond adequately to treatment [25]. NSS also corresponds with negative symptoms and cognitive impairment in people with schizophrenia [26,27,28]. Furthermore, evidence suggests that audio-visual hallucinations might be predicted by higher scores of integrative functions subscale, these findings possibly being based on changes in the gray matter volume of the right inferior parietal lobule a structure of the sensorimotor network and left superior temporal gyri as being a region of the auditory network [29]. NSS is much more common in patients with childhood-onset and adolescent-onset schizophrenia than in patients with adult-onset schizophrenia, suggesting that NSS may be markers of neurodevelopmental abnormalities in schizophrenia, as evidenced by the lack of suppression of primitive reflexes with cortical maturation [16,30,31]. NSSs are linked to a higher risk of psychosis in the general population, and motor abnormalities in adolescents at risk for psychotic disorders may predict potential psychotic disorder conversion [17,32,33]. According to meta-analyses, up to 73% of schizophrenia patients outperform healthy persons on average NSS measures [27].

Clinically, NSS may be used to monitor the illness course or to identify people who are predisposed to schizophrenia, but they are not currently monitored in clinical practice. NSS are not typically recognized in schizophrenia patients in a routine clinical context, but they can be discovered on a regular basis by clinical assessment, but even so, there is no widely accepted NSS evaluation instrument [8,34,35,36]. A considerable amount of data supports the concept that cortico-cerebellar-thalamic-cortical circuit (CCTCC) disruption underpins both intrinsic and drug-induced motor disorders [14,37,38].

There are three distinct types of qualitative spontaneous movements in schizophrenia: (1) neurological soft signs, (2) abnormal involuntary movements, and (3) catatonic symptoms. Based on current diagnostic standards, these three spontaneous movements are classified as independent and nonoverlapping categories, despite their complicated underlying etiological and pathophysiological causes [12,14,39,40].

A decrease in the activity of the enzymes involved in oxidative stress defense might constitute an intermediate phenotype in schizophrenia, as recent research correlates the NSS scores of first-episode psychosis or schizophrenia patients with a reduction of glutathione peroxidase activity, which also have the similarity of the correlation with the onset of schizophrenia and the lack of influence of the neuroleptic treatment on glutathione peroxidase activity [41].

### Aim of the Present Study

The interest in NSS research in patients with schizophrenia has grown substantially in recent years, owing in part to advances in brain imaging technology. However, only a fraction of the questions about the origin of NSS and its relationship to schizophrenia symptoms have been answered; there is still an ongoing discussion in the research community over the character of states (NSS expression correlated with symptomatology severity) versus trait (NSS regarded as endophenotypes) of NSS. The goal of the current study is to fill in information gaps about the significance of correlating NSS with morphological brain abnormalities and with the disease stage in the hopes that, in the future, patients with schizophrenia will receive more individualized treatments.

## 2. Methods

### 2.1. Screening and Selection Process

We performed a literature search to identify studies reporting on NSS in schizophrenia between January 2013 and January 2023 in the ScienceDirect and PubMed digital databases, by using the following terms: (neurological soft signs) AND (schizophrenia) for PubMed; {neurological soft signs schizophrenia} for ScienceDirect. In addition, we examined references from all articles included for additional relevant publications. To have a better picture of the knowledge on this subject, we also included studies that used magnetic resonance imaging (MRI) to correlate with the presence of NSS.

### 2.2. Exclusion Criteria

**Figure 1:**
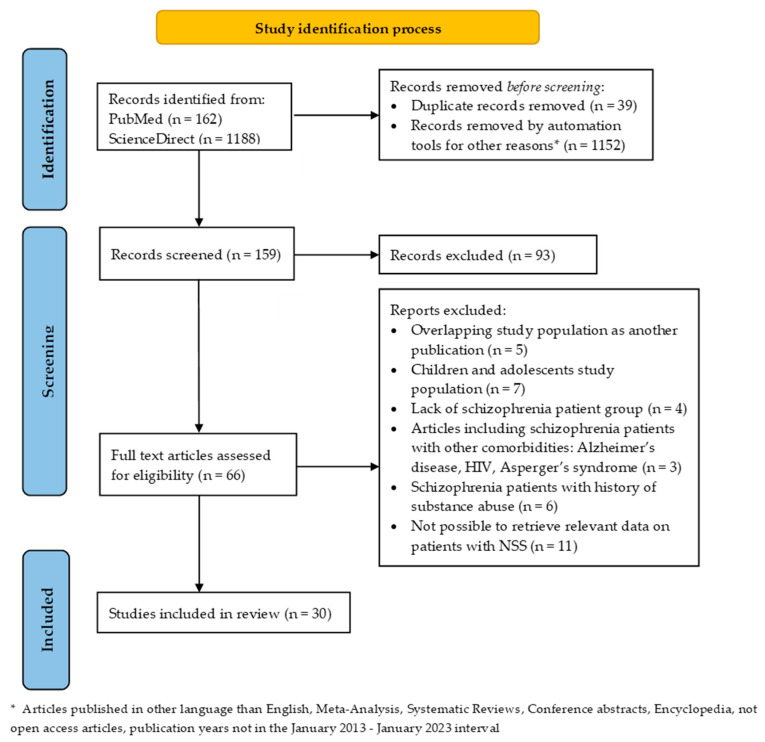
Flowchart of the study identification process.

We excluded the following: the literature on children and adolescents, studies that did not use a specific instrument for the measurement of NSS, studies including subjects with neurological or genetic diseases, intellectual disabilities or other comorbidities, studies that included patients with a history of substance abuse, articles published in languages other than English, Meta-Analysis, Systematic Reviews, Conference abstracts, articles that were not open access. The steps of the study identification process are presented in Figure 1. by following the Preferred Reporting Items for Systematic Review and Meta-Analysis (PRISMA) Extension guidelines for Scoping Reviews (PRISMA-ScR) [42].

We made the choice of excluding articles in which the authors clearly stated the inclusion of schizophrenia patients with alcohol abuse, based on the well-known interaction of alcohol with the central nervous system and thus influencing the expression of NSS, particularly those related to sensory integration, motor coordination, and other aspects of cognitive functioning. In addition to having a negative impact on cognitive and motor functioning, several studies found that patients with schizophrenia who had a history of alcohol abuse exhibited more neurological soft signs compared to patients without such a history, suggesting that alcohol may exacerbate the severity of NSS in individuals with schizophrenia. Overall, alcohol consumption can exacerbate NSS in individuals with schizophrenia, potentially making symptoms more noticeable and impairing daily functioning [43,44].

We further divided the selected articles into two categories. The first included either longitudinal or cross-sectional articles that presented data on NSS scores in correlation with symptomatology or were based on the comparison of NSS scores of patients with schizophrenia in comparison to healthy control groups or groups of patients’ relatives. The second set of publications includes studies that used MRI to correlate the existence of NSS with various brain structural alterations in people with schizophrenia. For comparison, we extracted data from each study regarding the total number of participants, the mean age of the participants, the illness stage, the tools used for assessing NSS, symptomatology and side effects of medication, if provided, and the type of antipsychotics and the mean daily dose. As stated in the PRISMA-ScR Checklist, items 15, 16, 22, and 23 are not applicable for scoping reviews, thus the “Risk of bias across studies” and “Additional analyses” such as subgroup analyses and meta-regression were not included in the present study [42].

## 3. Results

The initial search conducted in the two electronic databases revealed a total of 1350 publications. Once duplicates (*n* = 39) were removed by the authors and another 1152 records removed by automation tools, 159 titles and abstracts were examined, from which 66 remaining studies were further reviewed in full-text. For the present review, thirty (*n* = 30) articles were included, 12 of them included MRI structural evaluation and 4 studies with a longitudinal design [45,46,47,48].

An increased interest in the NSS has led to the creation of several different structured instruments for the evaluation and rating of NSS. Although there are many scales designed to rate NSS, in the present review, the following scales were used: Neurological Evaluation Scale (NES) [49]—in 12 studies, Cambridge neurological inventory (CNI) [50]—in 6 studies, Heidelberg scale [8]—in 10 studies, Krebs scale [34]—in 2 studies. Even though each of these instruments measures NSS, the degree of overlap between them is a particularly important factor in data reporting and can be the explanation for findings that cannot be replicated due to the use of different evaluation methods [36].

The total number of patients included in the identified manuscripts ranged from 20 [48] to 205 [51]. Eight studies [45,47,52,53,54,55,56,57] included patients that at the time of inclusion did not receive any antipsychotic regime, with only one study [53] that included antipsychotic—naive patients only. Dosages, where given, are hardly comparable between studies due to the different ways of reporting and different stages or strategies of the treatment. Nevertheless, most authors reported that they did not interfere with the choice of the antipsychotic regime, nor any modification of the daily dose was made due to the results of the NSS evaluation.

The endophenotype hypothesis of familial association is supported by the observation that the distribution of NSS is relatively similar between individuals with schizophrenia and their unaffected first-degree relatives [18]. In the present review, seven studies that included a group of first-degree relatives of patients with schizophrenia were identified [51,52,58,59,60,61,62]. Most studies included in the present review used different symptomatology assessment scales to correlate the severity of the clinical picture with the presence of NSS. The most used instruments were the Positive and Negative Syndrome Scale (PANSS) [63], and the Brief Psychiatric Rating Scale (BPRS) [64]. In addition, in order to highlight whether the neuroleptic treatment influences the NSS scores, several authors included, in view of the statistical correlations, scientifically approved scales for extrapyramidal adverse reactions such as the abnormal involuntary movements scale (AIMS) [65], the Barnes Akathisia Rating Scale (BARS) [66], the Simpson-Angus Scale (SAS) [67].

For each study, the main data relevant to the NSS correlation with sociodemographic data, symptomatology, and medication were summarized according to Item 21: Synthesis of Results of the PRISMA-ScR [42] in Table 1 and the main findings of articles that used structural imaging methods were presented in Table 2.

## 4. Discussion

### 4.1. NSS Correlation with Symptoms, Demographic Characteristics, and Illness Stage

Most studies compared schizophrenia patients to healthy subjects. Some authors also included patients with mental illnesses other than schizophrenia, or schizophrenia spectrum disorder [52,53,55,68,69,75] to assess the validity of their results. Zhao et al. [69] included in their study patients with schizophrenia, major depression, and bipolar disorder, and for comparison they used a control group of healthy subjects. The authors concluded that both patients with schizophrenia and patients with bipolar disorder showed more NSS total scores than healthy controls or patients with major depression. Although NSS total score and subscales could not differentiate patients with schizophrenia from those with bipolar disorder, by comparing the NSS scores to those of healthy controls, patients with schizophrenia exhibited more disinhibition signs, while patients with bipolar disorder displayed more sensory integration signs. A 2018 meta-analysis [82] of 18 studies that included a comparative analysis of schizophrenia patients, patients with bipolar disorder, and healthy controls, suggests small evidence of increased NSS scores in patients with schizophrenia than those with bipolar disorder, with only motor coordination tasks being a significant marker of differentiation. Participants’ age, gender, duration of illness, or the age of symptoms onset were not found to have a moderating effect on overall NSS scores. On the other hand, in cross-sectional research by Focseneanu et al. [68], patients with schizophrenia, obsessive compulsive disorder (OCD), and patients with an association of the two diagnoses were included. The authors concluded that although there was no statistically significant difference in the motor coordination and sensory integration subscales between the three groups, the sequencing subscale of complex motor acts scores differentiated patients with schizophrenia from those with OCD.

Compared to other symptoms analyzed during the evaluation, negative symptoms of schizophrenia were covered by some authors more thoroughly [46,47,70,76], suggesting connections between motor signs and negative symptoms, as well as positive relationships over time between overall NSS ratings and negative symptoms. The same conclusion was reached in a 2017 review [7] where the authors identified 14 published from 1996 to 2017 articles that focused on the correlation between the presence of NSS and clinical symptoms, especially negative symptoms. In cross-sectional research, Herold et al. [71] divided the schizophrenia patients and control group into three age groups, suggesting a correlation between NSS with aging, duration of illness, and a decline of positive symptoms. However, as the author suggested, a longitudinal approach to the study design was more appropriate to further strengthen the conclusion of NSS fluctuation in parallel with aging. In a subsequent study conducted by the same group [72], the authors strengthen the association between NSS and aging by demonstrating that patients with chronic schizophrenia have higher NSS scores, while in parallel, theory of mind and logical memory scores decline with age. Moreover, several other studies [57,70] included in the present review suggested an association between NSS and age, while others found no statistically significant evidence [77,78]. On this issue, the literature presents contradictory results. In several systematic reviews and meta-analyses, the authors [17] suggest a linear elevated trajectory of patients with schizophrenia and NSS in time, in contrast with a U-shaped pattern of NSS in healthy controls, while others found no evidence for age to be a significant moderator of NSS [27,83]. These contradictory results can be due to several causes, such as the difference in the average age of the groups, duration of illness (DOI), the period in which the patients received the appropriate treatment, the stage of illness, and the design of study performed (cross-sectional vs longitudinal Bachmann, Silkeudinal).

An important argument to consider NSS as a trait or endophenotype in schizophrenia is the prevalence of NSS in patient’s first-degree relatives with comparable scores of SI and MC as the patients. A significant number of studies have demonstrated that relatives have higher scores than healthy controls, and the closer the relatives (twins [20,84], siblings), the closer the NSS scores are to the values of schizophrenia patients [7,17,18]. A meta-analysis by Neelam K. et al. [18] included seven reports on NSS values in patients with schizophrenia in comparison to their first-degree relatives and healthy controls.

In the present review, it was also conclusive that individuals with schizophrenia exhibited NSS more frequently than healthy subjects, thus NSS seems to be dispersed in a way that is compatible with genetic links among individuals with schizophrenia and their first-degree relatives. The authors pointed out increased NSS values in people with schizophrenia compared to HC and their relatives, but also increased levels of NSS in first-degree relatives compared to HC.

In research conducted by Chen et al. [51], the authors examined NSS scores of healthy relatives of early onset schizophrenia (EOS) and adult-onset schizophrenia (AOS) patients in comparison with a group of healthy participants. One of their findings was that non-psychotic relatives of EOS and AOS patients scored considerably higher on all NSS subscales than healthy controls. Furthermore, EOS patients had higher scores than AOS patients on the SI and MC NSS subscales, suggesting that assessing NSS might be an important tool in discriminating against the neurological changes of the early onset of schizophrenia. In another research [58], although the authors found NSS scores to be comparable in first episode psychosis patients to their siblings, and higher than the healthy control group, there was no correlation reported between extrapyramidal symptoms with NSS and cognitive impairment in the siblings or healthy control group. In an article by Galindo et al. [61], the authors aimed to address this issue with a structural imaging analysis of the default mode network (DMN) abnormalities in schizophrenia patients, their non-psychotic relatives, and healthy controls. A conclusion of correlation between NSS and DMN connectivity was suggested, with less connectivity between the caudate nucleus and core brain networks as contributing to the presence of NSS. In line with previous reports, patients scored higher than their relatives and control. Moreover, while patients showed increased connectivity correlated with NSS in the left fusiform gyrus and posterior cingulate cortex than their healthy relatives, the relatives presented higher connectivity correlated to NSS scores than healthy controls in anterior prefrontal cortex analysis.

### 4.2. NSS Correlation with Medication and Side-Effects

Extrapyramidal side effects, such as acute or tardive dyskinesias, Parkinsonism, or akathisia, can be caused mostly by typical antipsychotic drugs. However, extrapyramidal adverse effects could not adequately explain NSS in clinical trials, thus, at this moment it is considered that neuroleptic medication does not induce NSS, implying that therapy may not be a major moderator [7], as suggested by studies that underlined the presence of NSS in patients with schizophrenia, prior to antipsychotic medication [85,86,87]. Whereas antipsychotic medication influences the presence of NSS, neither confirmation nor disapproval can be made, the same holds true for the antipsychotic class used or the combination of multiple antipsychotics. In general, the literature provides several hypotheses regarding the influence of treatment on NSS, such as a positive influence on NSS scores in patients with a first psychotic episode or a predictor of treatment resistance based on the NSS scores in first episode psychosis of drug-naive patients [14]. The effect of medication may be related to the stage of illness and the extent of NSS, which have been cited as predictors of medication response. At least two main groups of medicated patients appear to exist: one that improves clinically and in terms of NSS in parallel, and another that displays stable or worsening psychopathology and NSS. Despite the contradictory findings, it is likely that antipsychotic medications might have a protective effect on NSS scores in time [7]. Increased presynaptic dopamine activity sensitivity in the nigrostriatal pathway could be a potential mechanism for genetic susceptibility to movement disorders and schizophrenia. Imaging studies have revealed an increase in labeled dopamine accumulation in the striatum of drug-naive patients with schizophrenia, as well as in their healthy first-degree relatives, and basal ganglia volume loss correlated with NSS scores attributed to genetic factors, rather than to antipsychotic medication [88,89,90].

For the present review, the results of the studies included having conflicting conclusions on the treatment’s influence on NSS. Several authors [45,58,69,70,71,78,79] found no statistically significant correlation between the daily dose of antipsychotics and the NSS scores, while others suggested a link between the treatment and NSS scores in particular situations. For example, in one study [73], the authors concluded that a higher antipsychotic dose might be a reasonable predictor of treatment-resistant schizophrenia. In a cross-sectional imaging study [80] of first episode schizophrenia patients, the authors reported a partial correlation between CPZ and NSS mostly in NSS total score and MC subscale, but no correlation between CPZ and MRI measures, but iron loading measures of left-side basal ganglia correlated with extrapyramidal abnormalities and NSS. In a longitudinal study [46], Sambataro et al. concluded that NSS scores and parkinsonism improved with antipsychotic treatment 6 months after the remission or partial remission of acute psychotic symptoms.

### 4.3. NSS during Follow-Up

One study [46] including 96 schizophrenia patients with a mean duration of illness of 11.69 years (SD 10.31) and a mean age of 37.55 years (SD 10.39), evaluated the patients at baseline and after 7 months and concluded that NSS scores at baseline predicted the evolution of psychopathological symptoms. Changes in the PANSS positive symptoms were predicted by the Integrative Functions (IF) score, while motor coordination (MOCO) scores predicted PANSS negative symptoms scores on the follow-up. Moreover, the authors concluded that after the baseline evaluation, NSS total score improved with the antipsychotic treatment. In contrast to these findings, in a 2019 longitudinal study, Fountoulakis et al. [45], after evaluating 133 clinically stabilized patients with schizophrenia, with a mean age of 33.55 (SD 11.22) for over a 12-month period, found that NSS scores to be stable during the follow-up, except for sensory integration (SI) which became worse, although the overall symptomatology improved, and only the motor coordination (MC) scores were correlated with the clinical picture after 1 year, with no correlation reported at baseline. Thus, the author’s statement was the fact that NSS scores could not predict the clinical outcome.

A research team [47] included in their longitudinal study 123 first-episode schizophrenia patients with a mean duration of illness of 14.28 months (SD = 17.45) and subdivided the group based on scores of the PANSS in a “Prominent Negative Symptoms” group and a “Non-Prominent Negative Symptoms” group, and for comparison, a healthy control group of 62 patients was included. After the baseline, 6 months, and 1 year evaluation of the patients, NSS scores showed a decreasing trend in parallel with the overall clinical symptoms, but the “Prominent Negative Symptoms” group presented higher scores, especially on the motor coordination subscale (MC) in comparison to “Non-Prominent Negative Symptoms” group. As expected, both groups had higher NSS total scores than the HC group, thus this group was only evaluated at baseline. Although it did not have a control group and the study design was cross-sectional, another article [70] had similar conclusions on increased NSS scores, especially in patients with predominantly negative symptoms.

The state characteristic of NSS was emphasized in an MRI longitudinal study [48], in which twenty first episode schizophrenia patients with a mean age of 25.6 (SD 7.2) were examined after a mean duration of 13.8 months. Although the authors did not report any statistically significant correlation between the PANSS total score and NSS scores after the patients were subdivided into two groups (“persisting NSS subgroup”, “decreasing-NSS subgroup”), they hypothesized that alterations in local network properties of a cortical-subcortical-cerebellar circuit are the basis of the hypothesis of classifying NSS as a phenotype underlining the neurodevelopment of schizophrenia. A meta-analysis [83] that included 17 longitudinal studies published between 1992 and 2012 concluded that, despite significant methodological contrasts of the studies included, there is further evidence that patients with a remitting course of schizophrenia experience a greater decline in NSS ratings as their psychopathological symptoms improve than do patients with a non-remitting course of schizophrenia, in which these patients display either a stable or increasing trend of NSS, as a further meta-analysis suggests [7].

### 4.4. NSS and Imaging Findings

Studies based on magnetic resonance imaging (MRI) showed increased NSS scores in schizophrenia patients that are related to brain morphological alterations in structures such as the thalamus [57,75,79], caudate nucleus [61,62,75,77,80], putamen [48,62,80], globus pallidus [77,80], cerebellum [48,76,77,79], and the brainstem [78]. A study [79] found that individuals with chronic schizophrenia experienced progressive cognitive deterioration, as well as rises on the NSS subscales of MC and SI. Furthermore, the patient group’s NSS total scores were found to be significantly associated with lower GM in the right lingual gyrus, left parahippocampal gyrus, left superior temporal gyrus, left thalamic medial dorsal nucleus, and left posterior cerebellar lobe. Additionally, cerebral volume loss was correlated with the subscales MC, CMA, and RLSPO.

In a study [78] that included first-episode schizophrenia patients, greater NSS scores and higher scores on subscales MC, CMA, and hard signs were substantially associated with brainstem volume reduction. In comparison, no substantial relationships were identified between volumetric measures and scores on the subscales RLSPO, and SI. These associations pointed to regionally localized morphometric alterations, mostly in the pons and midbrain, rather than to global atrophy of the brainstem.

Higher sensory and spatial NSS were negatively correlated with local gyrification index (LGI) changes in the left precentral gyrus and the left precuneus in a study [81] of (LGI) in patients with recent-onset schizophrenia. Furthermore, higher motor NSS were linked to LGI changes, primarily in the supramarginal gyrus, the right superior parietal lobe, and the left superior temporal gyrus. Cortical thickness was found to have a negative relationship with NSS total scores in the left paracentral lobule and precuneus. In the left postcentral gyrus, negative associations between cortical thickness and higher NSS ratings on the HS subscale were found. Moreover, a correlation was established between cortical thickness in the left postcentral gyrus and higher NSS values on the subscale RLSPO. Higher ratings on the SI subscale were positively correlated with cortical thickness in the left middle temporal and supramarginal gyrus. These results demonstrate that cortical thickness may be a very sensitive marker and a potential indicator of a distinct process in schizophrenia individuals with NSS. Functional connectivity anomalies of the default model network (DMN) associated with NSS in schizophrenia were investigated in cross-sectional research [61]. The functional and structural anomalies related to NSS may be supported by aberrant connectivity between the caudate nucleus and other regions that make up the DMN. NSS ratings varied considerably between groups, with patients having higher scores. In comparison to controls, the connectivity study of individuals with schizophrenia showed significant hyperconnectivity in the fusiform gyrus, insular and dorsolateral prefrontal cortex, inferior and middle frontal gyri, middle and superior temporal gyri, and posterior cingulate cortex. In addition, healthy relatives of schizophrenia patients exhibited hyperconnectivity in comparison to controls in the supramarginal and dorsal posterior cingulate cortices [16]. Moreover, schizophrenia patients display aberrant cerebro-cerebellar functional connectivity with increased connectivity in somatomotor and default mode networks, and indications of poor functional connectivity between these networks, without overlapping the default mode hyper-connectivity with those found to be hyper-connected within the same default mode network [91].

### 4.5. Strengths and Limitations

This review must be considered considering several limitations. First, the clinical heterogeneity of the study population varies among a sample size of patients, the method of healthy controls selection, the duration of untreated psychosis, inconsistencies between inclusion and exclusion criteria, the type of first-degree relatives, scales used, and clinical course of the disease, diversity in items and scales used to evaluate dyskinesia and parkinsonism, may be a source of inconsistency. Most studies had a relatively small sample size, and most studies were conducted in patients who were at that time receiving antipsychotic medication with only one study [53] that included antipsychotic-naive patients only.

Few studies performed on NSS and brain abnormalities with MRI data acquisition strategies, and different methods of morphometric measurements used in various studies add up to the present limitations. Although currently there are numerous NSS assessment scales, each with strengths but also limitations in their complexity, it would be preferable to reach a consensus in the future on a single valid NSS assessment tool that would represent a “gold standard”. Due to a weak overlap of the evaluating items included in each scale, as was concluded in a 2021 review [36] on seven of the most used NSS scales, another limitation is the interpretation of the comparative results of studies that are similar to the point of view of the included patients but different from the point of view of the NSS scale used. To enhance the understanding of the variations in NSS scores and to better grasp the impact of each symptom change on the overall clinical presentation, it might be advisable to employ multiple NSS instruments during future research. This would entail making more comparisons and drawing more comprehensive conclusions.

Regarding the limitations of the present study design, we did not rigorously weigh studies quality as in a systematic review, and a broader more inclusive research question was used. Another limitation is the inclusion of other schizophrenia spectrum disorders or other psychotic disorders in addition to schizophrenia. The primary strength of the current scoping review is a detailed mapping of knowledge on NSS in schizophrenia, not only including known underlying relationships between NSS and the symptomatology of schizophrenia but also methodological limitations in conducting related studies. Furthermore, it is our hope that by disseminating the research findings of the previous decade, the current paper will lay the groundwork for a future full systematic review.

## 5. Conclusions

Since neurological soft signs can be detected swiftly, consistently, and inexpensively [92], they could represent a future useful tool in routine clinical settings to identify potential clues of whether an individual had advanced along the neuro-developmental trajectory to schizophrenia. It is essential to highlight that currently, international guidelines do not support the use of NSS scales as a tool in the diagnosis of schizophrenia or in the physician’s choice of selecting a targeted therapy. The present review contains articles on NSS in schizophrenia with at least partly contradictory findings. Although there are arguments in follow-up studies that report fluctuations regarding the expression of NSS in parallel with the evolution of symptoms, thus arguing for a state perspective, on the other hand, the fact that NSS are present at the onset of psychotic symptoms, and are not influenced by antipsychotic medication, these arguments speak in favor of a trait perspective. Putting these arguments together, we can state that both theories present arguments in their favor and are involved at the same time, but future solid evidence is needed to conclude this ongoing debate. Most studies showed higher NSS scores in schizophrenia patients than healthy controls, with a negative correlation between NSS subscores, motor coordination, and complex motor tasks. Other areas included disinhibition (D), sensory integration (SI), motor coordination (MC), and sequencing of complex motor acts (SCMA). Patients with a remitting course exhibit the highest change in their scores as measured by decreasing NSS ratings in parallel with symptomatology. Total NSS scores, motor coordination, motor sequencing, and sensory integration were higher in individuals with schizophrenia compared to their unaffected first-degree relatives, while cognitive performance was lower. Superior temporal lobe activation was increased, and basal ganglia and inferior frontal cortex activation were decreased in schizophrenia patients, these findings being related to the prevalence of NSS. Moreover, imaging studies revealed decreased white matter volume in the middle temporal and cerebellar areas, as well as diminished grey matter volume in the precentral and inferior frontal gyri, thalamus, and other brain regions. Further studies on larger patient groups incorporating various imaging techniques are thus required to better understand how these variables interact with and add to NSS.

In schizophrenia, the presence of NSS displays a pattern of hereditary association that is consistent with the state of an endophenotype for the condition, however the results are based on a limited number of studies therefore a homogenous population to demonstrate whether NSS are an endophenotype of schizophrenia is needed, otherwise there will be a limitation in the strength of the evidence. To what extent can existing psycho-pathological tests be improved through the detection of NSS in patients at risk, further prospective studies are needed. Current evidence is insufficient to guide the true nature of the NSS. Additionally, there is little evidence to precisely support how the presence of NSS can be used as a predictor of the development of the clinical picture in schizophrenia.

## Figures and Tables

**Table 1:** Studies showing a correlation of NSS with the clinical course of disease; Both cross-sectional and longitudinal studies included.

Author/Year/Country	Patients/Healthy Controls/Relatives	Age Mean (SD)	Follow-Up	Illness Stage	NSS Assessment Instrument	Clinical Course Assessment Instrument	Medication Mean (SD)	Side Effects/Parkinsonism	NSS Results Mean (SD)	Main Findings
[45] Fountoulakis/2019/ Greece	Patients (*n* = 133)	33.55 (11.22)	Longitudinal t0 baseline t1 12 months follow-up	stabilized	NES	*PANSS* PANSS-P t0:16.22 (5.81) t1:14.20 (4.61) PANSS-N t0:18.17 (7.37) t1: 20.02 (6.71) PANSS-G t0:26.20 (7.03) t1:26.16 (6.87)	6.25 (6.05) mg/day haloperidol equivalents. 35 of the patients were not under medication at baseline.	SAS ESRS UKUS	NES-SI t0:4.29 (3.09) t1:5.12 (3.04) NES-MC t0:3.89 (2.84) t1:3.44 (2.59) NES-CMA t0:6.67 (3.50) t1:6.71 (3.35) NES-OS t0:4.68 (3.51) t1:5.18 (3.39) NES Total t0:19.54 (9.48) t1:20.45 (8.87)	NSS have been constant over a 12-month period except for SI, which deteriorated significantly despite improvements in the rest of the clinical picture.
[46] Sambataro/2020/Germany	Patients (*n* = 43)	37.55 (10.39)	Longitudinal t0: baseline t1: 28 Weeks	partial remission	Heidelberg	*PANSS* PANSS-P t0:15.32 (7.32) t1:11.48 (5.68) PANSS-N t0:18.46 (8.27) t1:14.76 (5.71) PANSS-G t0:36.44 (12.62) t1:30.04 (9.0) PANSS-T t0:70.23 (23.39) t1:55.81 (17.49) BPRS Total score t0:39.23 (13.94) t1:30.06 (9.58)	t0:18.09 (9.89) OLZ t1: 18.75 (8.25) OLZ	AIMS t0:0.67 (1.5) t1:0.44 (1.14) SAS t0:2.74 (1.9) t1:1.83 (1.91) BARS t0:1.0 (1.25) t1:0.62 (1.0)	MC t0:8.18 (3.84) t1:6.51 (4.16) COMT t0:3.65 (2.23) t1:2.48 (2.34) IF t0:2.86 (1.56) t1:2.39 (1.46) RLSPO t0:2.72 (2.6) t1:6 2.2 (2.3) HS t0:3.25 (1.6) t1:2.67 (1.86) Total t0:20.19 (8.01) t1:15.97 (9.02)	With antipsychotic therapy, NSS, parkinsonism, and catatonic symptoms improved and displayed a substantial decline >6 months after the acute psychotic episode. NSS scores at baseline predicted PANSS positive and negative symptoms change at >6 months follow-up
[47] Chan/2015/China	PNS (N = 29) NPNS (N = 116) HC (N = 62)	22.34 (4.06) 21.69 (3.768) 21.16 (1.89)	Longitudinal T1–baseline T2–6 months T3–12 months	first episode schizophrenia	CNI	*PANSS* PANSS-T 60.93 (13.91) 42.16 (14.91) PANSS-N 21.79 (5.22) 9.48 (3.51) PANSS-P 12.34 (5.44) 10.98 (5.05)	14 patients receiving no treatment	SAS AIMS BARS	CNI-Total 7.13 (3.17) 5.61 (3.07) 2.76 (2.21) CNI-MC 3.68 (1.98) 2.64 (1.88) 1.34 (1.28) CNI-SI 2.26 (1.50) 1.99 (1.40) 0.74 (1.13) CNI-D 1.19 (0.97) 0.98 (0.93) 0.68 (0.65)	PNS had significantly more motor coordination impairment and total NSS than NPNS.
[51] Chen/2019/Taiwan	Total (N = 205) EOS (N = 82) AOS (N = 123) FS (N = 134) HC (N = 243)	41.89 (9.58) 36.85 (9.91) 43.59 (9.19) 56.52 (14.97) 42.10 (10.95)	cross-sectional	stabilized	NES 2 Raters	DIGS	NA	NA	NES Total EOS 9.95 (6.91) AOS 7.58 (5.44) C 1.43 (1.69) NES-SI EOS 1.43 (1.71) AOS 0.59 (0.85) C 0.15 (0.43) NES-MC EOS 0.93 (1.69) AOS 0.38 (0.54) C 0.02 (0.13) NES-CMA EOS 2.17 (1.84) AOS 1.99 (1.69) C 0.43 (0.68) NES-OS EOS 4.26 (2.97) AOS 3.10 (1.87) C 0.43 (0.68)	EOS patients had more severe SI and MC scores than AOS patients. On the SI and MC NSS subscales, there were substantial differences between non-psychotic relatives of EOS and AOS patients.
[52] Caldani/2017/ France	HC (*n* = 48) FS (*n* = 41) SZ (*n* = 49) UHR (*n* = 24)	25 (5) 28 (9) 28 (6) 21 (3)	cross-sectional	stabilized symptoms	Krebs Scale	*PANSS* PANSS- P 13 (5) PANSS- N 18 (6) PANSS- G 31 (9) PANSS-T 61 (19) BPRS	41 patients treated with anti- psychotics; 7 patients received no treatment	SAS AIMS	NSS total 5 (4) 6 (4) 12 (8) 11 (5)	Patients had more intrusive saccade eye movements than siblings or controls. In smooth pursuit, patients with high NSS ratings made more intrusive saccades than patients with low NSS scores and controls
[53] Chan/2018/China	HC (N = 39) Schizotypy (N = 39) UHR (N = 39) Patients (N = 39)	18.36 (2.08) 19.31 (2.54) 18.03 (5.01) 18.00 (3.75)	cross-sectional	first episode schizophrenia	CNI	*PANSS* PANSS-P 11.17 (4.49) PANSS-N 15.17 (6.62) PANSS-G 24.33 (7.02) SIPS	antipsychotic-naïve	not specified	CNI-SI 1.44 (1.37) 1.51 (1.34) 2.36 (1.35) 2.03 (1.37) CNI-MC 0.79 (1.36) 0.74 (1.12) 0.67 (1.13) 2.92 (2.17) CNI-D 0.74 (0.99) 0.92 (0.98) 0.92 (1.46) 1.00 (1.03) CNI-Total 2.97 (2.32) 3.18 (2.35) 3.95 (2.48) 5.95 (3.41)	UHR patients had a greater prevalence of NSS than healthy controls. Individuals with first-episode schizophrenia had a greater frequency of NSS than HC and UHR.
[54] Fountoulakis/2019/ Greece	HC (*n* = 110) Patients (*n* = 120)	33.38 (10.14) 32.79 (11.11)	cross sectional	NA	NES	NA	6.07 (6.12) mg/day haloperidol equivalents 33 patients receiving no medication.	NA	NES-SI 0.12 (0.40) 4.33 (3.18) NES-MC 0.20 (0.54) 3.93 (2.77) NES-CMA 0.54 (0.83) 6.61 (3.53) NES-OS 0.19 (0.61) 4.53 (3.49) NES Total 1.05 (1.42) 19.40 (9.44)	The patients had greater NES scores than controls, but sporadic cases of schizophrenia had lower MC scores than familiar cases.
[55] Fritze/2019/Germany	Total (*n* = 105) schizophrenia (*n* = 94) schizoaffective (*n* = 6) Schizotypal (*n* = 5)	38.02 (12.07)	cross-sectional	partial remission	Heidelberg	*PANSS* PANSS-P 16.1 (7.34) PANSS-N 17.49 (7.91) PANSS-G 36.46 (11.22) PANSS-T 70.04 (21.65) CGI-S 3.94 (1.05)	17.68 (10.49) OLZ equivalent 8 patients with no antipsychotic treatment 63 patients receiving one antipsychotic 34 patients receiving two antipsychotics	AIMS SAS BARS	MC 8.01 (4.12) IF 2.71 (1.57) COMT 3.47 (2.24) RLSPO 2.83 (2.6) HS 3.06 (1.74)	The dose of antipsychotic medication had no significant impact on NSS values.
[58] Cuesta/2018/Spain	Patients (*n* = 50) FS (*n* = 21) HC (*n* = 24)	25.5 (5.7) 24.9 (6.6) 23.2 (5.7)	cross-sectional	first- episode psychosis	NES 3 Raters	CASH positive 0.48 (0.64) negative 0.68 (0.83) disorganization 0.39 (0.57) mania 0.46 (0.81) depression 0.68 (0.93)	428.15 (299.87) CPZ	SAS 4.20 (3.82) 1.57 (1.66) 0.58 (1.13) BARS 0.48 (0.86) 0.00 (0.00) 0.00 (0.00) BFCRS 2.26 (3.89) 0.33 (0.96) 0.13 (0.61)	NES total 13.42 (6.44) 8.57 (6.09) 5.75 (4.46) NES-SI 2.5 (1.82) 1.71 (2.02) 1.33 (1.37) NES-MC 2.04 (2.26) 0.81 (1.03) 0.33 (0.86) NES-CMA 2.86 (2.24) 1.38 (1.68) 1.13 (1.26) NES-OS 6.02 (3.79) 4.66 (4.21) 2.95 (2.61)	In all three subgroups, higher NSS scores were associated with worse cognitive performance. Extrapyramidal symptoms were associated with cognitive impairment in FEP patients but not in unaffected relatives or healthy controls. In FEP patients, the NES total score was associated with poor attention performance.
[59] Feng/ 2020/ China	Patients (*n* = 86) FS (*n* = 86) HC (*n* = 86)	30.79 (8.17) 33.92 (10.07) 30.01 (7.47)	cross-sectional	patients with a remission course.	CNI	BPRS 2 Raters 25.23 (5.53)	104.41 (68.84) CPZ	NA	CNI-D 0.65 (1.08) 0.53 (0.98) 0.58 (0.94) CNI-MC 5.12 (4.01) 4.16 (2.84) 2.41 (1.85) CNI-SI 2.86 (2.94) 1.14 (1.43) 1.43 (1.44) CNI Total 8.63 (5.98) 5.84 (4.44) 4.42 (2.78)	In all subscales except disinhibition and motor coordination, patients had more NSS than first-degree relatives. NSS revealed significantly greater abnormalities in FDR than in controls.
[60] Schäppi/2018/Switzerland	HC N: 29 FS N: 34 Patients (N = 43)	40.86 (14.38) 42.74 (15.73) 37.98 (11.37)	cross-sectional	NA	NES	*PANSS* PANSS-T 71.14 (16) PANSS-P 17.98 (6.28) PANSS-N 18.14 (5.15)	NA	AIMS Total 0.14 (0.52) 1.15 (2.36) 1.91 (2.7)	NES Total 3.90 (3.59) 11.65 (8.18) 13.74 (11.75) NES-SI 1.14 (1.19) 2.94 (2.15) 3.58 (5.12) NES-MC 0.55 (0.78) 1.79 (1.82) 2.60 (2.84) NES-CMA 1.03 (1.96) 3.21 (2.72) 2.60 (2.96) NES-OS 1.14 (1.43) 3.71 (3.44) 4.88 (4.73)	The patients’ relatives had higher NSS ratings than healthy controls. Complex fine motor function was normal in relatives but impaired in patients.
[68] Focseneanu/2015/ Romania	Schizophrenia patients (*n* = 26) Schizophrenia + OCD Patients (*n* = 17)	aged up to 26 years	cross-sectional	recent relapse requiring hospitalization or corrective therapeutic intervention	NES	SCID-I/P (the Structured Clinical Interview for DSM IV Axis, I Disorders	NA	NA	NES Total 6.65 (5.04) 10.00 (4.89) NES-MC 0.80 (0.98) 1.05 (1.29) NES-SI 1.50 (1.55) 2.23 (1.32) NES-CMA 2.34 (1.85) 4.64 (2.08) NES-OS 2.00 (1.93) 2.05 (1.98)	NSS scores distinguished patients with schizophrenia associated with OCD from those with schizophrenia, with variations most noticeable in the subscale of CMA.
[69] Zhao/2013/China	Patients (*n* = 30) HC (*n* = 30)	26.20 (5.78) 26.60 (5.97)	cross-sectional	NA	CNI 2 Raters	2 Raters PANSS-T 50.87 (12.74)	316.36 (252.61) CPZ	NA	CNI-MC 2.03 (1.88) 0.77 (1.10) CNI-SI 1.23 (1.07) 0.77 (0.82) CNI-D 1.77 (1.45) 0.80 (0.81) Total NSS 5.03 (2.88) 2.33 (1.81)	Schizophrenia patients had more NSS signs than healthy subjects. Antipsychotic dose did not influence NSS total and subscales scores.
[70] Petrescu/2022/ Romania	Patients (*n* = 99)	30.6 (10.4)	cross-sectional	Mixed (non-predominantly negative symptoms patients and Patients with predominantly negative symptoms)	NES	*PANSS* PANSS-P 21.6 (6.06) PANSS-N 21.4 (6.32) PANSS-G 41.8 (8.66) PANSS-T 84.8 (16.8)	Typical and atypical antipsychotics 424 (219) mg CPZ	SAS 3.04 (2.01)	NES-SI 1.67 (1.52) NES-MC 2.08 (1.60) NES-CMA 3.12 (1.98) NES-OS 3.57 (2.62) NES Total 10.5 (5.50)	Patients with predominantly negative symptoms had higher overall NES and PANSS scores, needed higher daily doses of antipsychotic medications, and were hospitalized for extended lengths of time.
[71] Herold/2018/Germany	Patients (*n* = 90) HC (*n* = 60)	Y: 23.71 (3.18) M: 40.76 (5.85) O: 61.15 (7.46) Y: 21.60 (3.06) M: 42.41 (5.30) O: 58.00 (7.08)	cross-sectional	Chronic + Subchronic in three age groups (18–29 Young, 30–49 Middle, +50 Older)	3 Raters Heidelberg	BPRS (Patients) TOTAL Y: 41.53 (9.42) M: 39.82 (9.57) O: 34.85 (7.23) SAPS SANS AES (Patients) TOTAL Y: 25.14 (12.94) M: 27.41(10.80) O: 28.19(13.18)	Y: 726.00 (730.14) CPZ M: 730.42 (698.41) CPZ O: 577.42 (552.16) CPZ	NA	NSS Total (Patients) Y: 12.10 (8.56) M: 13.42 (10.82) O: 27.58 (15.36) NSS Total (HC) Y: 2.30 (1.64) M: 3.00 (2.40) O: 5.76 (3.73)	NSS increases with age in both schizophrenia patients and healthy controls, with the effect being more pronounced in the patients group. In patients with chronic schizophrenia, increased NSS scores are associated with illness duration, positive symptoms disorder, and apathy.
[72] Herold/2019/ Germany	Patients (*n* = 80) HC (*n* = 60)	43.36 (15.00) 47.52 (14.80)	cross-sectional	Chronic + Subchronic	Heidelberg	BPRS Total = 38.60 (9.17) SAPNS AES Total = 27.05 (12.15)	718.15 (691.43) CPZ	NA	Total 17.96 (13.94) 4.40 (3.44) MC 7.00 (6.15) 1.73 (1.73) SI 2.71 (2.23) 0.85 (1.13) COMT 3.05 (2.61) 1.08 (1.21) RLSPO 3.56 (3.62) 0.37 (0.78) HS 1.73 (2.04) 0.38 (0.83)	ToM (theory of mind) overall scores and the subscores “questions” and “order” were significantly linked with NSS total score and NSS subscales. NSS scores were considerably higher in older individuals with chronic schizophrenia who also had severe neurocognitive deficits. There were no substantial correlations between CPZ equivalents and NSS scores.
[73] de Bartolomeis/2018/ Italy	TRS (*n* = 26) SZ (*n* = 29)	39.7 (11.7) 35.0 (9.0)	cross-sectional	stabilized symptoms	NES	PANSS 2 Raters CGI-S	547.8 (210.4) CPZ (mg) 352.9 (217.3) CPZ (mg)	AIMS BARS NRS	NES-SI 5.6 (2.8) 3.6 (2.4) NES-MC 4.7 (2.9) 3.1 (2.1) NES-CMA 5.7 (3.5) 5.3 (3.3) NES-OS 11.5 (5.4) 6.9 (2.6) NES total 27.5 (12.1) 18.8 (6.6)	The more meaningful predictors of TRS diagnosis were higher SI and OS subscale scores, greater disease severity, and higher antipsychotic doses. NSSs were more severe in TRS patients than in SZ patients.
[74] Iasevoli/2018/Italy	TRS (*n* = 28) ARS (*n* = 32)	39.79 (11.31) 35.09 (9.02)	cross sectional	stabilized	NES	*PANSS* PANSS-P 19.41 (5.49) 18.51 (6.09) PANSS-N 26.61 (5.46) 22.56 (5.51) PANSS-G 49.26 (9.91) 45.34 (9.86) PANSS-T 96.03 (16.43) 86.72 (17.63)	CPZ 539.93 (214.51) 369.45 (215.41)	NA	NES Total 27.14 (11.74) 18.19 (6.72)	NSS is suggestive of TRS being a distinct subtype of schizophrenia with its own neurobiology and clinical course, possibly related to abnormal neurodevelopment.

**Abbreviations**: AES = Apathy Evaluation Scale; AIMS = Abnormal Involuntary Movement Scale; ARS = Antipsychotic Responder Schizophrenia; AOS = Adult-Onset of Schizophrenia; BARS = Barnes Akathisia Rating Scale; BFCRS = Bush-Francis Catatonia Rating Scale; BPRS = Brief Psychiatric Rating Scale; CGI-S = Clinical Global Impression–Severity; CMA = Complex Motor Acts; CNI = Cambridge Neurological Inventory; COMT = Complex Motor Tasks; CPZ = Chlorpromazine Equivalents mg; D = Disinhibition; DIGS = Diagnostic Interview for Genetic Studies; EOS = Early-Onset of Schizophrenia; ESRS = Extrapyramidal Symptom Rating Scale; FES = First-Episode Schizophrenia; FS = Patient’s First degree relatives; HC = Healthy Controls; HS = Hard Signs; IF = Integrative Functions; M = Middle age; MC = Motor Coordination; NA = Not Applicable; NES = Neurological Evaluation Scale; NPNS = Non-Prominent Negative Symptoms; NRS = Neurological Rating Scale for extrapyramidal symptoms; NSS = Neurological Soft Signs; O = Older age; OCD = Obsessive-Compulsive Disorder; OLZ = Olanzapine Equivalent mg; OS = Other Signs; PANSS = Positive and Negative Syndrome Scale (P = Positive, N = Negative, G = General, T = Total); PNS = Prominent Negative Symptoms; RLSPO = Right/Left and Spatial Orientation; SAPS = Scale for the Assessment of Positive Symptoms; SAPNS = Scales for the Assessment of Positive and Negative Symptoms; SAS = Simpson Angus scale; SI = Sensory Integration; SIPS-D = Structured Interview for Prodromal Symptoms—Disorganized symptoms; SIPS-G = Structured Interview for Prodromal Symptoms—General symptoms; SIPS-N = Structured Interview for Prodromal Symptoms—Negative symptoms; SIPS-P = Structured Interview for Prodromal Symptoms—Positive symptoms; TRS = Treatment Resistant Schizophrenia, SZ = responder schizophrenia; UHR = Ultra-High Risk for psychosis; UKUS = Udvalg for Kliniske Undersøgelser Scale; Y = Young age.

**Table 2:** Comparative structural imaging studies.

Author/Year/Country	Patients/Healthy Controls/Relatives	Age	Illness Stage	NSS Assessment	Clinical Course Assessment	Medication	Structural Assessment	Region of Interest	NSS Results	Main Finding
[48] Kong/2023/Germany	Patients (*n* = 20) [NSS decreasing subgroup (*n* = 10) NSS persisting subgroup (*n* = 10)] HC (*n* = 20)	25.6 (7.2) 24.1 (3.5)	First episode + follow-up after a mean of 13.8 months	Heidelberg	PANSS	baseline 549.3 (271.3) CPZ follow-up 443.8 (137.7) CPZ	T1- weighted magnetization-prepared rapid gradient echo (MP-RAGE)	Whole Brain	Decreasing NSS (*n* = 10) NSS baseline 15.9 (3.9) NSS Follow-up 7.2 (3.5) Persisting NSS (*n* = 10) NSS baseline 14.6 (9.2) NSS Follow-Up 14 (9.5)	Compared to the NSS-decreasing subgroup, the NSS-persisting subgroup displayed more widespread local alterations at follow-up affecting frontal and temporal cortices, the insula, the putamen, and the cerebellum
[56] Gay/2017/France	Patients (*n* = 41)	25.8 (6.0)	first episode	Krebs scale	PANSS	16 patients with no treatment 25 Low dosage of anti- psychotics 522 (414) mg CPZ	T1- weighted images	Cortex CSF	Total score 10.6 (6.3)	Anterior cingulate cortex (ACC) morphology’s impact on cognition is moderated by ventricle/sulcal enlargement, meaning that greater enlargement correlates with worse cognitive function. NSS are associated with executive impairments and handedness and correlates with ACC morphology.
[57] Viher/2022/Switzerland	HC (*n* = 42) Patients (*n* = 41)	39.3 (13.7) 37.9 (11.6)	NA	NES	PANSS-P 18.2 (6.3) PANSS-N 18.2 (5.2) PANSS-T 71.7 (17.1)	90% received antipsychotic treatment. 419.0 (362.9) CPZ	Diffusion tensor imaging Diffusion-weighted image	Whole Brain	NES total 3.7 (3.7) 12.2 (10.9) NES-SI 1.1 (1.2) 2.5 (2.6) NES-MC 0.6 (1.2) 2.2 (2.6) NES-CMA 0.9 (1.8) 3.0 (2.9) NES-OS 1.0 (1.4) 4.6 (4.6)	Patients had decreased fractional anisotropy (FA) values in various white matter regions, particularly the corpus callosum and anterior corona radiata, compared to HC. The total score of the NES scale was not associated with FA values
[61] Galindo/2017/Spain	HC (*n* = 35) FS (*n* = 23) Patients (*n* = 27)	36.60 (8.0) 41.39 (10.3) 37.60 (7.0)	clinically stable for the last 6 months	NES	PANSS	atypical antipsychotics	T1-weighted images default mode network (DMN)	Medial prefrontal cortex Posterior cingulate cortex Precuneus Anterior cingulate cortexes Angular gyruses Inferior parietal lobes Dorsolateral prefrontal cortex Angular gyruses	NES-MC 1.16 (1.05) 2.54 (2.55) 3.83 (2.2) NES-SI 1.18 (0.93) 1.67 (0.92) 3.03 (2.28) NES-SCMA 0.84 (1.22) 4.29 (1.55) 4.31 (1.81) NES-OS 1.5 (1.66) 2.42 (2.0) 5.79 (6.44) Total NES 4.68 (3.35) 10.92 (3.87) 16.97 (6.44)	Left caudate nucleus connectivity correlated significantly with NSS Schizophrenia patients and their unaffected relatives have greater NSS scores than controls.
[62] Kong/2022/ China	HC (*n* = 60) FS (*n* = 25) Patients (*n* = 62)	27.20 (6.21) 27.12 (7.26) 27.39 (6.37)	First-episode	CNI	NA	CPZ 329.44 (166.12)	T1-weighted MP-RAGE	Whole Brain	CNI-MC 1.50 (1.42) 2.40 (2.36) 3.71 (2.34) CNI-SI 0.42 (0.77) 0.96 (1.17) 1.24 (1.36) CNI-D 1.00 (0.61) 1.28 (0.68) 1.00 (0.91) CNI Total 2.92 (1.98) 4.64 (3.24) 5.95 (3.16)	Similar grey matter characteristics were observed in IC-5 (superior temporal gyrus, inferior frontal gyrus, and insula network) and IC-10 (parahippocampal gyrus, fusiform, thalamus, and insula network) in unaffected siblings and healthy controls. NSS were negatively associated with aberrant grey matter covarying networks in schizophrenia patients (IC-1 and IC-5), unaffected siblings (IC-3), and healthy controls (IC-5), indicating distinct NSS-related grey matter covarying patterns in each of the three groups.
[75] Quispe/2020/Spain	Patients = 102	24.9 (4.8)	Schizophrenia spectrum psychoses after remission of acute symptoms.	Heidelberg	BPRS 37.5 (15.8)	CPZ 722.2 (429)	T1- weighted 3D magnetization prepared rapid gradient echo sequence (MP- RAGE)	All brain structures	Total 14.2 (6.7)	The motor subscores of NSS were negatively correlated with the right superior frontal gyrus, thalamus, right caudate, and left precentral gyrus.
[76] Cai/2021/China	HC (*n* = 49) SZ (*n* = 50)	27.45 (6.42) 27.68 (6.27)	NA	CNI	*PANSS* PANSS-P 9.72 (3.09) PANSS-N 11.38 (5.96) PANSS-G 20.70 (5.09) *SANS* SANS Alogia 1.78 (2.92) SANS Avolition 1.58 (2.32) SANS Anhedonia 2.72 (4.08) SANS Attention 2.38 (2.22)	CPZ mg 340.83 (174.32)	Cerebellar- cerebral resting- state functional connectivity	Whole Brain	CNI-MC 1.33 (1.28) 3.04 (1.93) CNI-SI 0.37 (0.70) 1.18 (1.38) CNI-D 1.18 (0.69) 1.90 (1.27) CNI Total 2.86 (1.93) 6.14 (3.30)	In schizophrenia patients, cerebellar-prefrontal resting-state functional connectivity was substantially associated with motor coordination deficits and negative symptoms.
[77] Hirjak/2019/Germany	HC (*n* = 37) Patients (*n* = 37)	34.08 (11.83) 34.41 (11.00)	Partial remission schizophrenia and schizoaffective disorder	Heidelberg	*PANSS* PANSS-P 16.03 (8.51) PANSS-N 16.35 (8.02) PANSS-G 35.62 (10.44) PANSS-T 68.00 (22.49) (BPRS)	15.81 (9.27) mg OLZ	Three measurements resting-state scan T1-weighted three-dimensional magnetization- prepared rapid gradient-echo (3D-MPRAGE) diffusion-tensor imaging	Whole Brain	MC 7.62 (3.73) SI 2.43 (1.44) COMT 3.08 (2.37) RLSPO 2.43 (2.18) HS 2.92 (1.72)	There were no significant differences in age, gender, or years of education between SCHIZO and HC patients. Frontocerebellar and frontoparietal networks correlated negatively with motor coordination scores in patients.
[78] Hirjak/2013/Germany	Patients (*n* = 21)	22.6 (3.6)	First-episode schizophrenia	Heidelberg	BPRS 24.0 (7.9) SAPS 21.8 (14.8) SANS 29.3 (21.3)	Atypical Antipsychotics 504.8 (327.6) CPZ	3D T1-weighted images	Brainstem	Total 12.5 (9.7) MC 5.2 (4.5) SI 1.8 (1.5) COMT 1.2 (1.6) RLSPO 1.0 (1.8) HS 2.0 (1.8)	Higher NSS scores, and particularly higher scores on the subscales of motor coordination, complex motor tasks, and hard signs, are correlated with structural modifications of the brainstem, predominantly in the pons and midbrain, rather than to general atrophy of the brainstem as a whole.
[79] Herold/2020/Germany	Patients (*n* = 49) HC (*n* = 29)	42.33 (13.93) 48.21 (13.56)	Chronic Subchronic	Heidelberg	3 RATERS BPRS TOTAL 38.08 (9.66) SAPNS AES 26.67 (11.96)	CPZ 638.37 (569.57)	T1-weighted magnetization prepared rapid gradient echo (MP-RAGE)	Occipital Lobe Frontal Lobe Lingual Gyrus Parahippocampal Gyrus Superior Temporal Gyrus Thalamus Cerebellum	Total NSS 16.90 (12.37) 3.79 (3.29) MC 6.67 (5.60) 1.69 (1.44 SI 2.51 (2.12) 0.52 (0.79) COMT 2.82 (2.51) 0.66 (1.17) RLSPO 3.47 (3.39) 0.48 (0.95) HS 1.61 (1.69) 0.45 (0.74)	No significant associations between CPZ NSS scores. NSS significantly correlated to cerebral volume loss in patients.
[80] Cuesta/2021/Spain	Patients (*n* = 48) HC (*n* = 23)	25.7 (5.8) 23.4 (5.9)	first episode	NES	CASH	CPZ mg 413.62 (280.65)	3D axial T1-weighted sequence shape analysis of subcortical structures	Left and right puta- men Globus pallidus Caudate nucleus Accumbens	NA	First episode (FEP) patients’ CPZ equivalent antipsychotic medication exposure did not exhibit any significant correlations in imaging measures. However, CPZ was found to have a substantial relationship with NES total scores in FEP. Significant shape changes occurred in the right accumbens of FEP patients with high scores on the tremor subscale and in the left caudate of FEP patients with high scores on the NES sensory integration subscale.
[81] Hirjak/2015/Germany	Patients (*n* = 33)	23.12 (4.24)	Recent-onset schizophrenia	Heidelberg	BPRS 24.57 (10.48) SAPS 20.78 (15.07) SANS 32.3 (21.25)	SAA 489.21 (310.28) mg CPZ	T1-weighted 3D magnetization prepared a rapid gradient echo sequence. (MP-RAGE) local gyrification index (LGI)	Cortex	Total NSS 13.57 (9.13) MC 6.18 (4.57) SI 1.72 (1.37) COMT 1.6 (1.76) RLSPO 1.24 (1.73) HS 1.78 (1.76)	Higher sensory and spatial NSS are negatively associated with LGI alterations in the left precentral gyrus and left precuneus. Higher motor NSS are associated with LGI alterations in the supramarginal gyrus bilaterally, the right superior parietal region, and the left superior temporal gyrus.

**Abbreviations**: AES = Apathy Evaluation Scale; BPRS = Brief Psychiatric Rating Scale; CASH = Comprehensive Assessment Symptoms and History; CMA = Complex Motor Acts; CNI = Cambridge Neurological Inventory; COMT = Complex Motor Tasks; CPZ = Chlorpromazine Equivalent dose mg; CSF = Cerebro-Spinal Fluid volume; D = Disinhibition; FE = First Episode psychosis; FS = Patient’s First degree relatives; HC = Healthy Controls; HS = Hard Signs; MC = Motor Coordination; SZ = Patients with Schizophrenia; NA = Not Applicable; NES = Neurological Evaluation Scale; NSS = Neurological Soft Signs; OLZ = Olanzapine Equivalents mg; OS = Other Signs; PANSS = Positive and Negative Syndrome Scale (P = Positive, N = Negative, G = General, T = Total); RLSPO = Right/Left and Spatial Orientation; SAA = Single Antipsychotic Agent; SANS = Scale for Assessment of Negative Symptoms; SAPS = Scale for the Assessment of Positive Symptoms; SAPNS = Scales for the Assessment of Positive and Negative Symptoms; SCMA = Sequencing of Complex Motor Acts; SI = Sensory Integration.

## Data Availability

Not applicable.
